# National Prociency Testing Result of CYP2D6*10 Genotyping for Adjuvant Tamoxifen Therapy in China

**DOI:** 10.1371/journal.pone.0162361

**Published:** 2016-09-07

**Authors:** Guigao Lin, Kuo Zhang, Lang Yi, Yanxi Han, Jiehong Xie, Jinming Li

**Affiliations:** 1 Beijing Hospital, National Center of Gerontology, Beijing, China; 2 Beijing Hospital, National Center for Clinical Laboratories, Beijing, China; 3 Beijing Hospital, Beijing Engineering Research Center of Laboratory Medicine, Beijing, China; 4 Graduate School, Peking Union Medical College, Chinese Academy of Medical Sciences, Beijing, China; University of South Alabama Mitchell Cancer Institute, UNITED STATES

## Abstract

Tamoxifen has been successfully used for treating breast cancer and preventing cancer recurrence. Cytochrome P450 2D6 (CYP2D6) plays a key role in the process of metabolizing tamoxifen to its active moiety, endoxifen. Patients with variants of the *CYP2D6* gene may not receive the full benefit of tamoxifen treatment. The CYP2D6*10 variant (the most common variant in Asians) was analyzed to optimize the prescription of tamoxifen in China. To ensure referring clinicians have accurate information for genotype-guided tamoxifen treatment, the Chinese National Center for Clinical Laboratories (NCCL) organized a national proficiency testing (PT) to evaluate the performance of laboratories providing CYP2D6*10 genotyping. Ten genomic DNA samples with *CYP2D6* wild-type or CYP2D6*10 variants were validated by PCR-sequencing and sent to 28 participant laboratories. The genotyping results and pharmacogenomic test reports were submitted and evaluated by NCCL experts. Additional information regarding the number of samples tested, the accreditation/certification status, and detecting technology was also requested. Thirty-one data sets were received, with a corresponding analytical sensitivity of 98.2% (548/558 challenges; 95% confidence interval: 96.7–99.1%) and an analytic specificity of 96.5% (675/682; 95% confidence interval: 97.9–99.5%). Overall, 25/28 participants correctly identified CYP2D6*10 status in 10 samples; however, two laboratories made serious genotyping errors. Most of the essential information was included in the 20 submitted CYP2D6*10 test reports. The majority of Chinese laboratories are reliable for detecting the CYP2D6*10 variant; however, several issues revealed in this study underline the importance of PT schemes in continued external assessment and provision of guidelines.

## Introduction

Tamoxifen is widely used as an anti-estrogenic drug for the treatment of estrogen receptor (ER)-positive breast cancer [1]. Clinical trials demonstrate that tamoxifen therapy can reduce breast cancer recurrences and improve patient survival rates [2, 3]. Tamoxifen is a prodrug that is metabolized mainly by hepatic cytochrome P450 2D6 (CYP2D6) into active metabolites [4]. 4-Hydroxytamoxifen (4OHT) and 4-hydroxy-N-desmethyltamoxifen (endoxifen), the two active therapeutic metabolites, exhibit 100-fold greater affinity to ERs and significantly greater potency in suppression of estrogen-stimulated cell proliferation compared to that exhibited by tamoxifen [5].

*CYP2D6*, the first cloned human-drug-metabolizing gene, is the most polymorphic drug-metabolizing gene, and is involved in the metabolism of up to 25% of commonly prescribed medications. The variant alleles of *CYP2D6* can substantially affect its enzymatic activity, with variations in activity divided into four classes: ultrarapid metabolizer (UM), extensive metabolizer (EM), intermediate metabolizer (IM), and poor metabolizer (PM). The CYP2D6*3, CYP2D6*4, CYP2D6*5, and CYP2D6*6 are the major null alleles found in Caucasians [6]. PM and IM patients have lower plasma concentrations of endoxifen and benefit less from tamoxifen therapy [7]. In contrast, the CYP2D6*10 (100C>T, rs1065852; 4180G>C, rs1135840) allele, with decreased enzymatic activity, has been found in 40–50% of Asians [8, 9]. A large sample study revealed that CYP2D6*10 is the most common allele (42.6%) in the Chinese Han population, followed by CYP2D6*1 (26.5%) [9]. Xu et al. reported that out of 152 Chinese women receiving tamoxifen therapy, patients with the CYP2D6*10/*10 genotype had a lower 4OHT plasma level and a worse disease-free survival rate [10]. Another study showed that the CYP2D6*10 variant affected the efficacy of combined tamoxifen citrate and testosterone undecanoate treatment in 230 infertile Chinese men [11]. Thus, genotyping of CYP2D6*10 can be used to optimize the selection [8, 10, 11] and dosing [12] of tamoxifen in eastern Asian patients.

In the era of personalized medicine, pharmacogenetic tests are used more frequently in Chinese clinical laboratories. To standardize and promote pharmacogenetic testing, the National Health and Family Planning Commission of the People's Republic of China has recently published guidelines on genetic testing technology for drug-metabolizing enzymes and drug targets [13]. The accuracy of genotyping tests is the foundation of clinical implementation of pharmacogenomics. Since 2014, the Chinese National Center for Clinical Laboratories (NCCL) has organized three Proficiency Testing (PT) programs for pharmacogenetic tests [14–16]. In 2015, to achieve inter-laboratory consistency and standardization of the results, the Chinese NCCL conducted a national PT scheme for the analysis of CYP2D6*10 allele. Here, we present the results of the scheme and evaluate the genotyping accuracy and clinical reports of CYP2D6*10 testing in China.

## Materials and Methods

### Preparation of genomic DNA samples

*CYP2D6* wild-type cell lines (GM17285, GM17216) and cell lines harboring the CYP2D6*10 allele (GM17240, GM16654) were purchased from Coriell Cell Repositories (Camden, New Jersey, USA). The consensus genotype of each cell line for the *CYP2D6* variants was confirmed by several assay platforms [17]. The cell lines were cultured according to methods described previously [18]. Genomic DNA was isolated from the cell cultures using the modified salting-out method [19]. The extracted genomic DNA was resuspended in tris-EDTA (TE) buffer (pH 8.0) at a concentration of 50 μg/mL. The solution was dispensed in 0.5 mL vials (0.1 mL each), labeled, and stored at -20°C.

### Validation of control samples

The CYP2D6*10 alleles in the quality control samples were confirmed by the NCCL reference lab using the Sanger sequencing method. Two pairs of primers were used: C100Tforward, 5′-TCGGTGTGCTGAGAGTGTCCT-3′, and C100Treverse, 5′-TGGTTTCACCCACCATCCAT-3′; G4180Cforward, 5′-AGCCAGGCTCACTGACG-3′, and G4180Creverse, 5′-AGGATGATCCCAACGAG-3′. Polymerase chain reactions (PCR) were performed in 50 μL volumes including 25 μL Gotaq Green Master Mix (Promega, Madison, USA), 0.2 μmol of the forward and reverse primers, and 100 ng of genomic DNA. Amplification was conducted using a Mastercycler (Eppendorf, Hamburg, Germany), by initial denaturation at 95°C for 5 min, followed by 35 cycles including denaturation at 95°C for 30 s, annealing at 55°C for 30 s, and extension at 72°C for 40 s. A final extension followed at 72°C for 5 min. The PCR products were purified and the sequencing was carried out using BigDye Terminator v3.1 Cycle Sequencing Kit (Applied Biosystems, Foster City, USA) on the ABI 3500DX Genetic Analyzer (Applied Biosystems) according to the manufacturer’s instructions.

### Organization of the PT

Our PT survey was open to any laboratories that offered or were intending to offer *CYP2D6* genotyping in China. Currently, a sample set of 10 challenges, selected according to sample characteristics and real clinical situations, is used in PT programs. A coded panel (*n* = 10) placed on ice (to prevent degradation) was delivered to the participating laboratories. Among the 10 samples (D1501-D1510), 4 were homozygous for *CYP2D6* wild *1/*1, 3 were heterozygous for CYP2D6*1/*10, and 3 were homozygous for CYP2D6*10/*10 (Table 1). The participating laboratories were asked to perform *CYP2D6* genotyping using their routine procedures. The results of the genotyping had to be submitted by laboratories within two weeks of the shipment date. They were also requested to provide information with regard to the testing method employed, the number of *CYP2D6* genotyping tests conducted per month, and the laboratory accreditation/certification status. After the PT survey, a general report summarizing the genotyping results and the common problems with clinical reporting was sent to each participant, along with individual suggestions in order to help the laboratories provide quality clinical genetic services.

**Table 1:** Genotyping results of the 28 participants.

Participant laboratory	Detection technique	D1501	D1502	D1503	D1504	D1505	D1506	D1507	D1508	D1509	D1510
		GM17240^a^	GM17285	GM16654	GM17240	GM16654	GM16654	GM17216	GM17285	GM17285	GM17240
Reference lab	Sanger sequencing	*1/*10	*1/*1	*10/*10	*1/*10	*10/*10	*10/*10	*1/*1	*1/*1	*1/*1	*1/*10
		100CT	100CC	100TT	100CT	100TT	100TT	100CC	100CC	100CC	100CT
		4180GC	4180GG	4180CC	4180GC	4180CC	4180CC	4180GG	4180GG	4180GG	4180GC
1–19^b^	Pyrosequencing	*1/*10	*1/*1	*10/*10	*1/*10	*10/*10	*10/*10	*1/*1	*1/*1	*1/*1	*1/*10
		100CT	100CC	100TT	100CT	100TT	100TT	100CC	100CC	100CC	100CT
		4180GC	4180GG	4180CC	4180GC	4180CC	4180CC	4180GG	4180GG	4180GG	4180GC
20–21	Sanger sequencing	*1/*10	*1/*1	*10/*10	*1/*10	*10/*10	*10/*10	*1/*1	*1/*1	*1/*1	*1/*10
		100CT	100CC	100TT	100CT	100TT	100TT	100CC	100CC	100CC	100CT
		4180GC	4180GG	4180CC	4180GC	4180CC	4180CC	4180GG	4180GG	4180GG	4180GC
22	Sanger sequencing	**Technical failure**^f^	*1/*1	*10/*10	*1/*10	*10/*10	*10/*10	*1/*1	*1/*1	*1/*1	*1/*10
			100CC	100TT	100CT	100TT	100TT	100CC	100CC	100CC	100CT
			4180GG	4180CC	4180GC	4180CC	4180CC	4180GG	4180GG	4180GG	4180GC
23	Sanger sequencing	*1/*10	*1/*10	*10/*10	*1/*10	*10/*10	*10/*10	*1/*10	*1/*10	*1/*10	*1/*10
		100CT	100CC	100TT	100CT	100TT	100TT	100CC	100CC	100CC	100CT
		4180GC	4180GC^g^	4180CC	4180GC	4180CC	4180CC	4180GC	4180GC	4180GC	4180GC
24–25	NGS^c^ and Sanger sequencing	*1/*10	*1/*1	*10/*10	*1/*10	*10/*10	*10/*10	*1/*1	*1/*1	*1/*1	*1/*10
		100CT	100CC	100TT	100CT	100TT	100TT	100CC	100CC	100CC	100CT
		4180GC	4180GG	4180CC	4180GC	4180CC	4180CC	4180GG	4180GG	4180GG	4180GC
26	ARMS^d^ and Pyrosequencing	*1/*10	*1/*1	*10/*10	*1/*10	*10/*10	*10/*10	*1/*1	*1/*1	*1/*1	*1/*10
		100CT	100CC	100TT	100CT	100TT	100TT	100CC	100CC	100CC	100CT
		4180GC	4180GG	4180CC	4180GC	4180CC	4180CC	4180GG	4180GG	4180GG	4180GC
27	ARMS	*1/*10	**Not reported**	**Not reported**	*1/*10	**Not reported**	*1/*10	**Not reported**	**Not reported**	**Not reported**	**Not reported**
		100CT	100CC	100CT	100CT	100CT	100CT	100CC	100CC	100CC	100CT
		4180GC	4180GC	4180CC	4180GC	4180CC	4180GC	4180GC	4180GC	4180GC	4180CC
28	SBE^e^	*1/*10	*1/*1	*10/*10	*1/*10	*10/*10	*10/*10	*1/*1	*1/*1	*1/*1	*1/*10
		100CT	100CC	100TT	100CT	100TT	100TT	100CC	100CC	100CC	100CT
		4180GC	4180GG	4180CC	4180GC	4180CC	4180CC	4180GG	4180GG	4180GG	4180GC

^a^ GM17240: Coriell cell line number.

^b^ 1–19, 20–21, and 24–25: Results of laboratories 1–19, 20–21, and 24–25 (have the same results and the same genotyping methods).

^c^ NGS: next-generation sequencing.

^d^ ARMS: amplification refractory mutation system.

^e^ SBE: single base extension.

^f^ Bolded text indicates genotyping errors.

^g^ Underlined text indicates allele errors.

### Statistical analysis

A board of assessors from the NCCL evaluated the results and at least 80% genotype accuracy for a dataset is considered proficient.

All data were conducted using MEDCALC software (MedCalc Software, Mariakerke, Belgium). Analytical parameters of detecting sensitivity and specificity were calculated. Confidence intervals of 95% (CI 95%) were also determined. Fisher's exact test was applied for comparison of rates. *P* < 0.05 was considered statistically significant.

## Results

### Sample validation

The genotype of each DNA sample extracted from cell lines provided by Coriell was confirmed by the NCCL laboratory using the Sanger sequencing method (Table 1).

### Participating laboratories and response

Twenty-eight laboratories, including 19 hospital laboratories and 9 commercial laboratories, participated in the PT. The names and locations of the 28 laboratories are presented in S1 Table. Two of the 28 participants were accredited according to ISO 15189, 2 laboratories were accredited according to ISO 17025, and another 2 laboratories were accredited by the College of American Pathologists (CAP). As stated by the laboratories, the average number of CYP2D6*10 genotyping tests performed per month was 179 (range: 0–900). Seven labs analyzed more than 100 samples per month.

All 28 participants submitted the results within the due date. Among them 3 laboratories presented two sets of results using two methodologies. Thus, a total of 31 completed datasets were generated.

### Analysis of results obtained from participating laboratories and *CYP2D6* genotyping assays

Genotyping results from the participants were compared with those of the reference laboratory (Table 1). In total, 25 (91.9%) participants reported all 10 challenges correctly (100% proficient), 1 laboratory made a genotype mistake (90% prficient), 1 laboratory made 4 mistakes (60% proficient), and 1 laboratory made 8 mistakes (20% proficient). Laboratory #23 wrongly identified 4180GG as 4180GC and thus reported CYP2D6*1/*1 as CYP2D6*1/*10 (sample D1502, D1507, D1508, and D1509). Laboratory #27 did not report the genotypes of samples D1502, D1503, D1505, D1507, D1508, D1509, and D1510. It was impossible for them to provide a correct assignment of genotype because their genotyping resulted in a discrepancy between 100C>T single nucleotide polymorphism (SNP) and 4180G>C SNP with these samples. In addition, Laboratory #27 wrongly reported CYP2D6*10/*10 as CYP2D6*1/*10 (sample D1506). Laboratory #22 reported a technical failure. The genotyping errors can be classified into two groups: false-positives (variant instead of wild-type) and false-negatives (wild-type instead of variant and not reported). In general, participants who carried out more tests per month performed well in this PT scheme. In contrast, each of the three laboratories that made genotyping mistakes processed less than 5 samples each month. Note that laboratory #27 had intended to offer *CYP2D6* genotyping. The genotyping accuracy of a laboratory was defined as correctly reported genotypes divided by expected genotypes of all samples. No significant difference in accuracy of detecting CYP2D6*10 was observed between hospital participants and commercial participants (178/190 vs. 89/90; *P* = 0.068), or between certified and non-certified laboratories (201/220 vs. 56/60; *P* = 0.494).

All laboratories employed laboratory-developed tests (LDTs). The most frequently used methodology was pyrosequencing. The performance of each genotyping technique was evaluated in terms of proficiency and testing parameters (Table 2).

**Table 2:** Proficiency results and analytical characteristics of genotyping methods used in the study.

Assay	No. of datasets	No. of datasets proficient at ^a^:			CYP2D6 genotype	
		100%	99–80%	< 80%	Sensitivity (%; CI 95%)	Specificity (%; CI 95%)
					Correct variant alleles/total variant alleles ^b^	Correct wild-type alleles /total wild-type alleles ^c^
Pyrosequencing	20	20	0	0	100;98.9–100 (360/360)	100;99.1–100 (440/440)
Sanger sequencing	6	4	1	1	94.4;88.3–97.9 (102/108)	98.5;94.7–99.8 (130/132)
NGS	2	2	0	0	100;90.2–100 (36/36)	100;91.9–100 (44/44)
ARMS	2	1	0	1	88.9;73.9–96.8 (32/36)	88.6;77.7–96.6 (39/44)
SBE	1	1	0	0	100;81.4–100 (18/18)	100;84.5–100 (22/22)
Total	31	28	1	2	98.2;96.7–99.1 (548/558)	96.5;97.9–99.5 (675/682)

^a^ 100% proficient: all genotypes reported correctly. 80%–99% proficient: 80%–99% of genotypes reported correctly. Not proficient: < 80% of genotypes reported correctly.

^b^ variant allele: 100T, 4180C.

^c^ wild-type allele: 100C, 4180G.

### Evaluation of clinical reports

**Fig 1:**
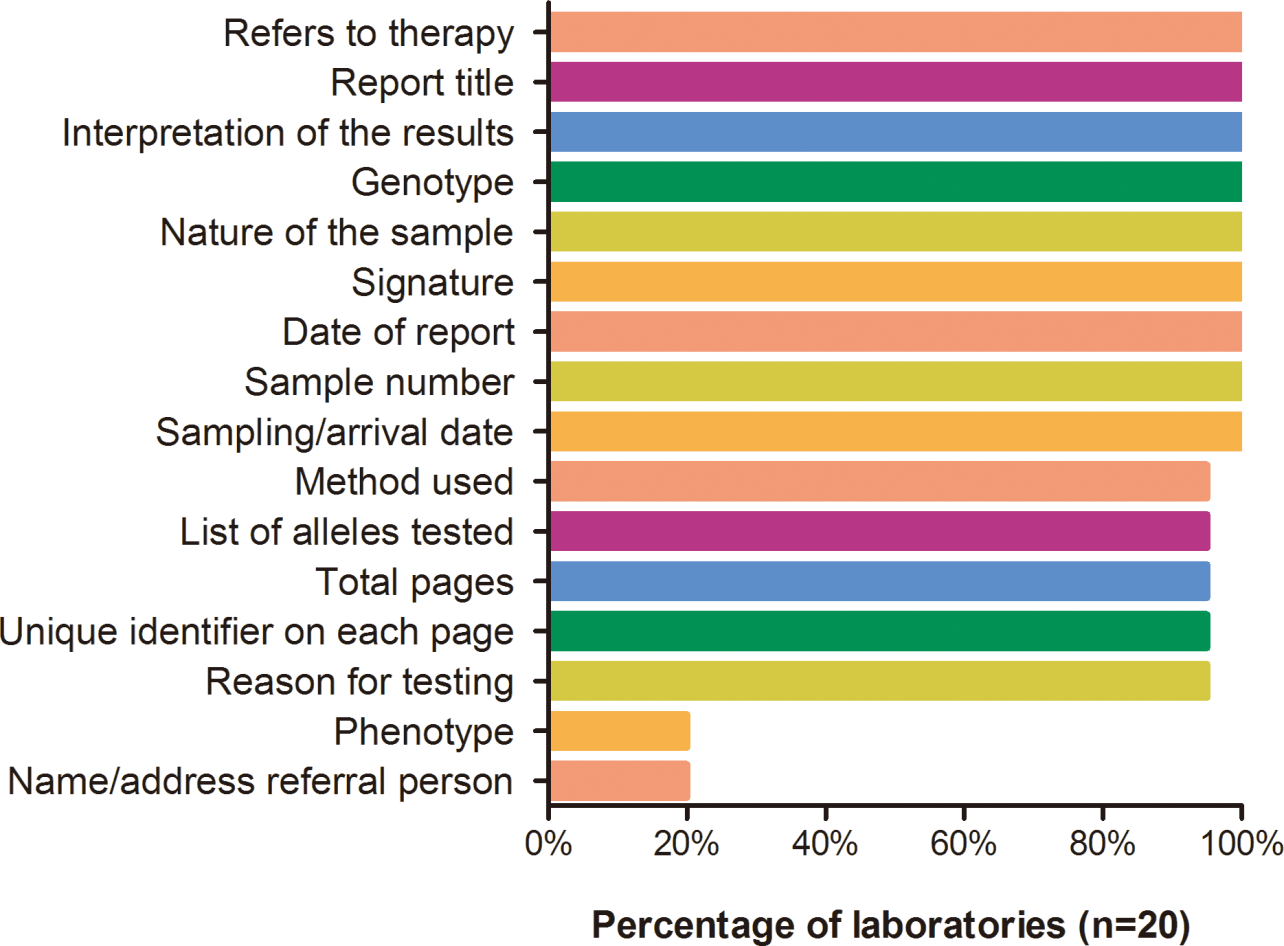
Reporting scores of the CYP2D6*10 proficiency testing scheme.

Each participant was asked to submit a clinical report for the first sample. As previously mentioned, 16 key items including genotyping result, phenotype, and interpretation of the results were evaluated [15]. For each item, one point was scored if the information was provided correctly. In total, 20 of the 28 laboratories submitted their reports and the mean score was 13.3 points (with a maximum of 16). Information regarding phenotype and consultants for the report was often not indicated (Fig 1). The clinical reports were not graded.

## Discussion

Substantial progress in the field of pharmacogenomics has been achieved in the past decades. Today, pharmacogenetic tests are widely used to select and optimize the prescription of drugs in individual patients. Goetz et al. showed that breast-cancer patients with reduced CYP2D6 enzyme activity are at increased risk of recurrence after tamoxifen therapy [20]. In addition, it was demonstrated that the CYP2D6*10 allele affects the efficacy of tamoxifen in Asian women receiving adjuvant tamoxifen therapy [8, 10]. Thus, the NCCL scientific board decided to introduce CYP2D6*10 variant analysis to the NCCL PT program of pharmacogenetics. In this study, we focused on the assessment of genotyping accuracy of CYP2D6*10 testing provided by Chinese laboratories. To improve the quality of reporting test results, the clinical reports were also reviewed for educational purposes.

The majority of the participating laboratories were 100% proficient in the NCCL CYP2D6*10 genotyping PT survey; however, two laboratories made serious mistakes. The identification of CYP2D6*10 was based on the correct detection of both 100C>T SNP and 4180G>C SNP. A genotyping error can lead to unfavorable outcomes if the wrong pharmacogenomics information was used to guide the selection of medication. For example, the false-positive results provided by lab #23 could prevent the patients from receiving tamoxifen therapy. In addition, the false-negative results produced by lab #27 could adversely affect the patients because they influence the administration of tamoxifen to IM carriers, for whom tamoxifen is suboptimal [21]. These laboratories are now aware of their mistakes. For laboratories that lack experience in pharmacogenetic testing, the use of well-characterized reference materials (RMs) for *CYP2D6* testing is necessary for test validation and quality control. These characterized RMs are available at the Coriell Cell Repositories. Note that the difference in genotyping accuracy between hospital and commercial participants was almost significant (*P* = 0.068). A possible explanation is that the hospital participants made more genotyping errors (12 errors) than the commercial participants (1 error) did.

All the participants employed LDTs, since none of the *CYP2D6* genotyping kits were approved by the China Food and Drug Administration (CFDA). Most of the testing technologies in this study demonstrated good performance in analytical sensitivity and specificity (Table 2). As in our previous PT schemes [14, 15], the utilization of pyrosequencing surpassed the use of other genotyping techniques in the present study. A subset of laboratories using Sanger sequencing and the amplification refractory mutation system (ARMS) showed inferior performance (Table 2). Nevertheless, other laboratories using the two technologies performed well, which indicates the problem was related to the single laboratory expertise. Before implementing a test in clinical care, it must be validated. Internal quality control samples are recommended for every test, especially for laboratories that lack experience in clinical genetic testing. Notably, two participants applied a targeted, capture-based, next-generation sequencing (NGS) test, which was conducted on an Illumina HiSeq 2500 system, without making any mistakes. In the near future, given decreasing sequencing costs, it is believed that all newborns will have their whole genome sequenced. Under such circumstances, the question of whether to opt for a pharmacogenomic testing should shift to how genetic information can be used for drug-prescribing guidance.

Complete and accurate reporting of clinical pharmacogenetic testing is of great importance. The overall quality of the reports received in this PT survey was good; however, eight laboratories did not submit the reports. The majority of laboratories provided essential information regarding the laboratory, patient, sample identifiers, genotyping results, method applied, and interpretation; however, the phenotype (EM or IM) was missing in most of the reports, leading to unclear reports. The assignment of phenotype is based on genotype, which is important for interpretation in regards to drug prescribing. At present, one of the major barriers that prevent the clinical implementation of pharmacogenomics is the dearth of well-accepted guidelines on how to conduct pharmacogenetic testing and reporting of test results. The aforementioned guideline [13] does not focus on the reporting of the pharmacogenetic tests.

Another PT experience for CYP2D6*10 genotyping, which was conducted by the CAP [22], showed a lower genotype concordance [95 responses/116 challenges, (81.8%)] when compared with ours [267 responses/280 challenges, (95.3%)]. The genotype concordance numbers obtained in the CAP survey were more likely a result of laboratories being able to submit star allele diplotypes based on their assay and variant panel, which included more alleles than just 100C>T and 4180G>C.

In summary, most Chinese laboratories testing for the CYP2D6*10 variant demonstrated good analytical performance. However, the poor genotyping results of two participants underline the necessity of continued external assessment in the pharmacogenetic testing community. Moreover, the NCCL PT programs [14, 15] and CAP PT [22] programs for pharmacogenetics have uncovered several issues, which calls for the framing of guidelines for both performing and reporting pharmacogenetic testing, to enhance the quality of laboratory results.

## Supporting Information

S1 Table The names and locations of the 28 laboratories that participated in the CYP2D6*10 PT survey. (DOCX)
