# Correction: National Prociency Testing Result of CYP2D6*10 Genotyping for Adjuvant Tamoxifen Therapy in China

**DOI:** 10.1371/journal.pone.0167740

**Published:** 2016-12-01

**Authors:** The PLOS ONE Staff

In the title of this article, the word “Prociency” should be “Proficiency.” The correct title is: National Proficiency Testing Result of CYP2D6*10 Genotyping for Adjuvant Tamoxifen Therapy in China. The correct citation is: Lin G, Zhang K, Yi L, Han Y, Xie J, Li J (2016) National Proficiency Testing Result of CYP2D6*10 Genotyping for Adjuvant Tamoxifen Therapy in China. PLoS ONE 11(9): e0162361. doi:10.1371/journal.pone.0162361. The publisher apologizes for the error.
