# Supplementary material for: National Prociency Testing Result of CYP2D6*10 Genotyping for Adjuvant Tamoxifen Therapy in China
Source: PLoS One. 2016 Sep 7;11(9):e0162361. doi: 10.1371/journal.pone.0162361 (PMC5015788; doi:10.1371/journal.pone.0162361)
Supplement: S1 Table — (DOCX) [file pone.0162361.s001.docx]

**S1 Table. The names and locations of the 28 laboratories participated in the *CYP2D6*10* PT survey.**

| **Laboratory** | **Address** |
| --- | --- |
| Department of pharmacy, Shanghai Changzheng Hospital | No.415 Fengyang Road,Shanghai,P.R.China |
| Deyi diagnostics | NO.8 xinjiekouwai street, Beijing, China |
| Department of pharmacy, Army General Hospital of PLA | No. 5 Dongsishitiao. South Gate Warehouse, Dongcheng District, Beijing, China |
| San valley diagnostics | NO.9 Guangyang street, Fangshan District, Beijing, China |
| Department of clinical laboratory, Zhongshan Hospital | 180 Fenglin Road, Shanghai, China |
| Guangzhou JINYU Medical Examination Center Co. Ltd | No.2429 Xingang East Road, Haizhu District, Guangzhou, Guangdong Province, China |
| Department of clinical laboratory, Huludao City Central Hospital | 15 Lianshan Street, Lianshan District, Huludao, Liaoning Province, China |
| Department of clinical laboratory, Taihe Hospital | No.32, People South Road, Shiyan City. Shiyan. Hubei Province, China |
| Department of pharmacy, Nanjing General Hospital of Nanjing Military Command | 305 Zhongshan East Road, Xuanwu District. Nanjing, Jiangsu, China |
| Department of pharmacy, Affiliated Hospital of Qingdao University Medical College | No.32, Jiangsu Road, Qingdao City, Shandong Province, China |
| Department of pharmacy, The First Affiliated Hospital of Xiamen University | No.55, Zhenhai Road, Siming District, Xiamen City, Fujian Province, China |
| Department of clinical laboratory, Shandong Cancer Hospital | No. 440, Jiyan Road. Jinan, Shandong ,China |
| Central laboratory, Second Hospital of Shanxi Medical University | 382 Wuyi Road, Taiyuan, Shanxi, China |
| Department of pharmacy, Shaanxi Provincial tumor hospital | No.3 Xincun Road, Xinghualing District, Taiyuan, Shanxi Province, China |
| Lifegene diagnostics | No.229 Taibai North Road , Xi'an, China |
| BGI clinical laboratories(Shenzhen) | No.11, Beishan Industrial Zone, Yantian District, Shenzhen 518083, China. |
| Department of pharmacy, Sichuan Provincial People's Hospital | #32 West Sec 2, 1st Ring Road, Qingyang District, Chengdu, China. |
| Department of pharmacy, The First Affiliated Hospital of Suzhou (Soochow) University. | No.188 Shizi Street,Suzhou, Jiangsu, China |
| Department of pharmacy, TianJin First Center Hospital | 24 Fukang Road, Nankai District, Tianjin, China |
| BGI clinical laboratories(Tianjin) | No.98 Ring Hebei Road,Tianjin Airport Economic Zone, Tianjin, China |
| Department of pharmacy, Weihai Municipal Hospital | No.70 Heping Road, Huancui District, Weihai 264200, China |
| Department of pharmacy, The Second Affiliated Hospital of Xi'an Jiaotong University | No.157, Xiwu Road, Xincheng Distict, Xi’an, China |
| Department of pharmacy, Xiking fourth military medical university hospital | 127 Chengle West Street, 710032, Xi'an, Shaanxi, China. |
| Department of pharmacy, Yantai Yuhuangding Hospital | East Road No. 20, Zhifu District, Yantai 264000, Shandong Province, China |
| 3G Biotech Co. Ltd | Building A5, Changsha Luvalley National Hi-Tech Industrial Development Zone, Lugu Road, Yuelu Dirstrict, Changsha, Hunan Province, P.R.China |
| 3G MEDICAL DIAGNOSTICS | Building A5, Changsha Luvalley National Hi-Tech Industrial Development Zone, Lugu Road, Yuelu Dirstrict, Changsha, Hunan Province, P.R.China |
| XIANGYA MEDICAL LABARATORY CENTRAL SOUTH UNIVERITY | 110 Xiangya Road, Kaifu District Changsha City, Hunan Province, China |
| Department of clinical laboratory, The First Affiliated Hospital of Chongqing Medical University | No.1 Youyi Road, Yuanjiagang, Yuzhong District, Chongqing, China. |
